# VILIP-1 Expression *In Vivo* Results in Decreased Mouse Skin Keratinocyte Proliferation and Tumor Development

**DOI:** 10.1371/journal.pone.0010196

**Published:** 2010-04-15

**Authors:** Jian Fu, Fang Jin, Jirong Zhang, Kathryn Fong, Daniel E. Bassi, Ricardo Lopez De Cicco, Divya Ramaraju, Karl-Heinz Braunewell, Claudio Conti, Fernando Benavides, Andres J. P. Klein-Szanto

**Affiliations:** 1 Department of Pathology, Fox Chase Cancer Center, Philadelphia, Pennsylvania, United States of America; 2 Cancer Biology Program, Fox Chase Cancer Center, Philadelphia, Pennsylvania, United States of America; 3 Southern Research Institute, Birmingham, Alabama, United States of America; 4 Department of Carcinogenesis, M.D. Anderson Cancer Center, Smithville, Texas, United States of America; University of Oldenburg, Germany

## Abstract

VILIP-1, a member of the neuronal Ca^2+^ sensor protein family, is able to act as a tumor suppressor in carcinoma cells by inhibiting cell proliferation and migration. In order to study the role of VILIP-1 in skin carcinogenesis we generated transgenic mice overexpressing VILIP-1 in epidermis under the control of the bovine keratin K5 promoter (K5-VILIP-1). We studied the susceptibility of FVB wild type and VILIP-1 transgenic mice to chemically mediated carcinogenesis. After 30 weeks of treatment with a two-stage carcinogenesis protocol, all animals showed numerous skin tumors. Nevertheless, K5-VILIP-1 mice showed decreased squamous cell carcinoma (SCC) multiplicity of ∼49% (p<0.02) with respect to the corresponding SCC multiplicity observed in wild type (WT) mice. In addition, the relative percentage of low-grade cutaneous SCCs grade I (defined by the differentiation pattern according to the Broders grading scale) increased approximately 50% in the K5-VILIP1 mice when compared with SCCs in WT mice. Similar tendency was observed using a complete carcinogenesis protocol for skin carcinogenesis using benzo(a)pyrene (B(a)P). Further studies of tumors and primary epidermal keratinocyte cultures showed that matrix metalloproteinase 9 (MMP-9) levels and cell proliferation decreased in K5-VILIP-1 mice when compared with their wild counterparts. In addition tissue inhibitor of metalloproteinase 1 (TIMP-1) expression was higher in K5-VILIP-1 keratinocytes. These results show that VILIP-1 overexpression decreases the susceptibility to skin carcinogenesis in experimental mouse cancer models, thus supporting its role as a tumor suppressor gene.

## Introduction

The chemical carcinogenesis model of skin cancer has been used extensively to better understand the sequence of events and the nature of lesions that appear during the course of the carcinogenesis process [1]. These studies resulted in innumerable findings and advances in the knowledge of tumor development, and furthered the concept that very small doses of carcinogen followed by hyperplasiogenic stimuli can result in cancer. Furthermore these findings collected during various decades, have been enhanced by more recent molecular data that established that many of the stages of carcinogenesis are due to a large extent to specific changes in oncogenes and tumor suppressor genes [2].

In a previous study, using a pair of low grade/high grade mouse SCC cell lines derived from the same primary tumor (CC4B/CC4A cells), we identified by differential display a gene product that was lost in invasive tumor cells [3]. This protein, VILIP-1 (gene name *VSNL1*), was established to be a member of the visinin-recoverin or neuronal calcium sensor (NCS) protein family [4], [5] and has been identified as having a role in human neurological disease and cancer [6]. The VILIP protein family contains 4 EF-hand calcium-binding motifs, and a consensus myristoylation site at its N-terminus. VILIP-1 modulates the levels of cyclic nucleotides by indirect or direct interactions with adenylyl and guanylyl cyclases [7], [8]. The modulation of cyclic adenosine monophosphate (cAMP) by VILIP-1 has been previously studied in nerve cells and has been demonstrated to be the mediator of the effect of VILIP-1 on cell proliferation, differentiation, and migration [5], [7], [8]. We reported that VILIP-1 is differentially expressed in murine skin tumors and cell lines of different degrees of aggressiveness and that transfection of two high grade mouse SCC lines with the *VILIP-1* cDNA, increased cAMP levels, leading to diminished MMP-9 activity together with a significant reduction in the invasive properties of the carcinoma cells [9].

In order to study the role of VILIP-1 during *in vivo* carcinogenesis we developed transgenic mice that express VILIP-1 in the epidermal basal layer. Although transgenic mice did not seem to have gross abnormalities, a more in depth analysis revealed that the tumor suppressive ability of this gene resulted in a decreased susceptibility to skin carcinogenesis.

## Results

### Generation and characterization of K5-VILIP-1 transgenic mice

**Figure 1:**
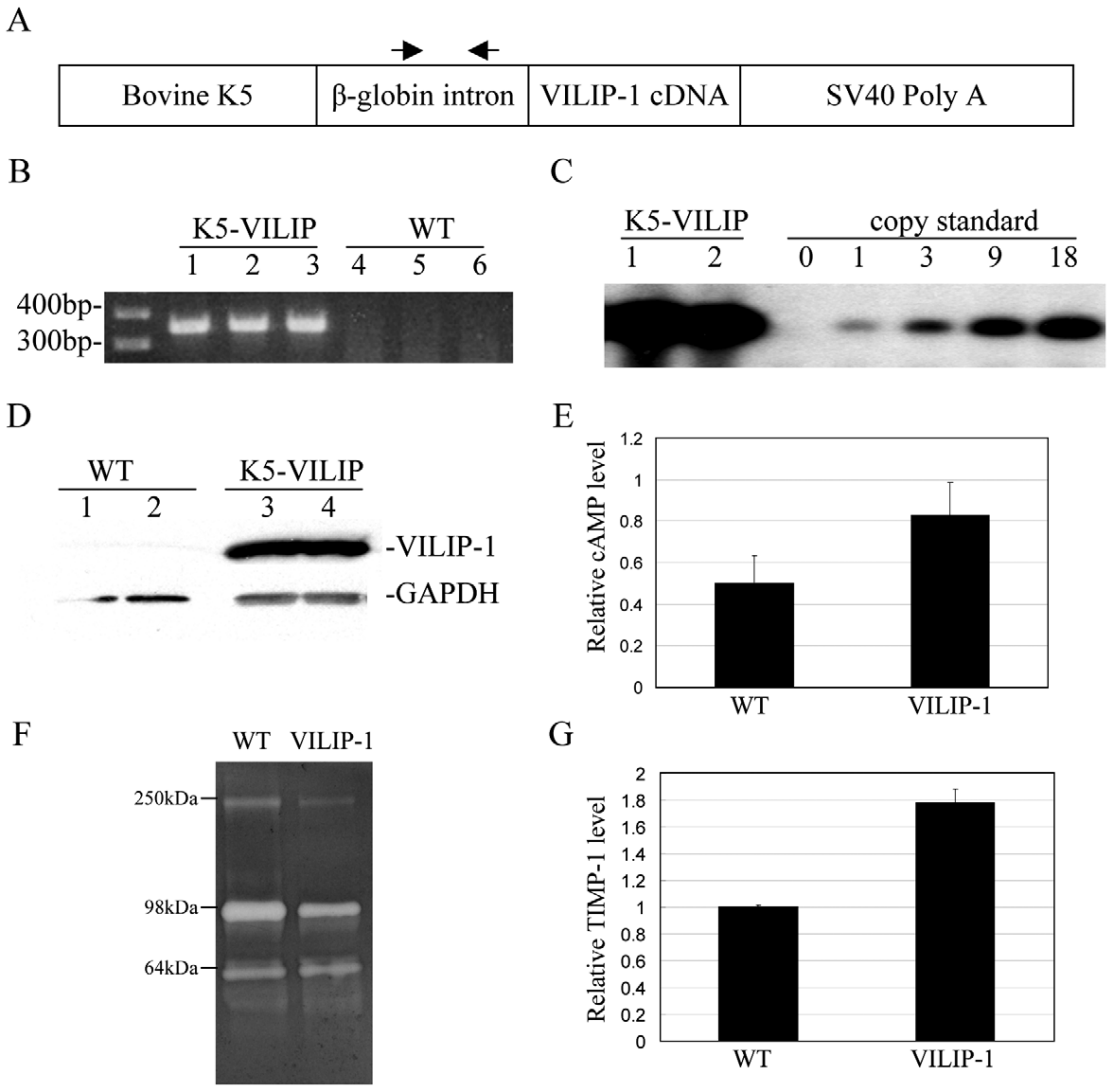
Generation of VILIP-1 transgenic mice. (A) K5-VILIP-1 construct. The two arrows above the β-globin intron box indicate the position of the specific primers used in PCR genotyping. (B) PCR from DNA extracted from WT and K5-VILIP-1 transgenic mouse tails, amplified with β-globin primers are shown. Lanes 1 to 3 correspond to three different K5-VILIP-1 transgenic mice showing positive band, lanes 4 to 6 correspond to three different non-transgenic mice, negative for the transgene band (300–400 bp). (C) Determination of the number of copies of the transgene by Southern blot analysis from 2 animals from the selected transgenic mouse line compared to copy standards as described in Material and Methods. (D) Western blot showing differential expression of VILIP-1 protein in primary keratinocyte cultures derived from WT (lanes 1 to 2) and transgenic mouse epidermis (lanes 3 to 4). (E) Intracellular concentrations of cAMP in primary epidermal keratinocytes derived from K5-VILIP transgenic mice (VP+) was higher than that from WT mice (VP-) (p = 1.2E-05). (F) Gelatinase zymography showing decreased MMP-9 activity of K5-VILIP-1 supernatant (+) derived from primary keratinocyte cultures when compared with their WT counterpart (−). Molecular weights corresponding to standards are shown at the left. (G) Relative TIMP-1 concentration in supernatant derived from K5-VILIP-1 transgenic (VP+) epidermal keratinocyte cultures was higher than that from WT (VP-) (p = 3.6E-09). The values have been normalized with respect to the WT.

To study the effects of VILIP-1 expression on the highly proliferative epidermal basal cells, the full-length human *VILIP-1* cDNA was placed under the control of the K5 promoter targeting VILIP-1 to the basal epidermal keratinocytes. The construct (Figure 1A) contains the bovine K5 promoter, followed by the first intron from rabbit β-globin to enhance the efficiency of transcription, the full-length *VILIP-1* cDNA and, finally the polyadenylation signal from SV-40. Two founders were produced and the one with the highest number of transgene copies was selected to generate a transgenic line (data not shown). The selected founder and its progeny were genotyped by PCR of genomic DNA using the primers amplifying a DNA segment of about 360 bp. A representative genotyping experiment is shown in Figure 1B.

Transgene copy number was assessed by Southern blot analysis. The transgenic line used for these experiments contained higher-than-18 copies of the transgene (Figure 1C). Transgene expression was confirmed by Western blot analysis of VILIP-1 protein expression. As source of proteins we used lysates from primary keratinocyte cultures obtained from newborn mice. VILIP-1 protein was readily detected in the keratinocytes from transgenic mice but not from wild-type counterparts (Figure 1D). Since VILIP-1 affects the levels of intracellular cAMP [3], the concentration of this cyclic nucleotide was measured in the primary epidermal keratinocytes derived from WT and K5-VILIP-1mice. Keratinocytes derived from the transgenic mice showed higher levels of intracellular cAMP than their WT counterparts (Figure 1E). Previous evaluation of VILIP-1 in transfected cells demonstrated decreased levels of MMP-9 [3]. Thus, we decided to evaluate this metalloprotease in primary keratinocytes. As depicted in Figure 1F, primary keratinocytes from K5-VILIP-1 transgenic mice showed lower levels of MMP-9 activity than keratinocytes derived from WT mice. Furthermore, we were able to demonstrate that at least part of this MMP-9 activity reduction in the transgenic-derived cells was due to an elevation of TIMP-1 expression in these cells when compared to WT keratinocytes (Figure 1G).

### Overexpression of VILIP-1 induces epidermal proliferative changes

**Figure 2:**
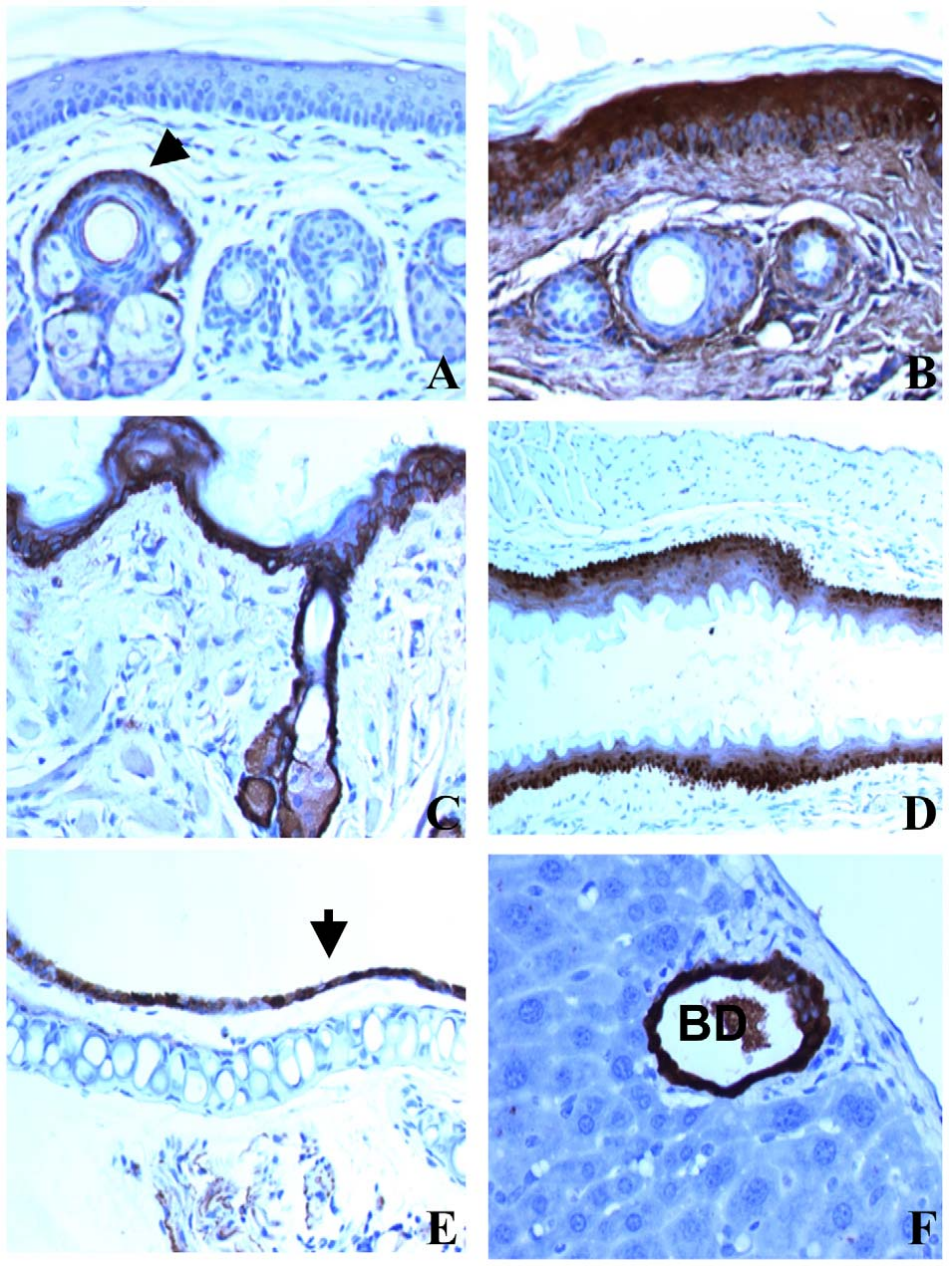
IHC analysis of VILIP-1 expression in mouse tissues. Normal tail skin of WT (A), and K5-VILIP-1 transgenic mouse (B). Note that a few basal keratinocytes (arrow), especially in the supra-sebaceous or infundibular portion of the hair follicle express VILIP-1 in the normal tail epidermis (A). In the transgenic epidermis there is a massive overexpression of VILIP-1 in practically every layer of the epidermis and adnexa (B). Similar overexpression is noted in the dorsal skin of K5-VILIP-1 transgenic mouse (C). Other VILIP-1-overexpressing epithelia in transgenic mice shown are: esophagus (D), bronchial epithelium (arrow) (E) and biliary duct epithelium (F). VILIP-1 immunohistochemistry counterstained with hematoxylin, X 200.

The gross and histological examination of the untreated skin of animals up to six months of age showed no deviations from normal. Nevertheless, immunohistochemistry (IHC) analysis of skin and other squamous epithelia showed that VILIP-1 was markedly overexpressed in these tissues. The overexpression was very obvious in the transgenic mice (compare Figures 2A and 2B) and could also be used to distinguish between transgenic and WT mice using small fragments of tail skin that were also used for PCR confirmation of the transgenic status. Minimal or absent VILIP-1 immunostain was noted in the tail and dorsal skin from WT mice. Occasionally a few basal keratinocytes showed mild immunostain in a focal pattern. Conversely, K5-VILIP-1 epidermis exhibited intense and diffuse immunostain in the basal and spinous layers (Figure 2B, C). Similarly VILIP-1, that was not seen or only marginally expressed in other epithelia from WT mice, e.g., oral epithelia, bronchial mucosa, and biliary duct epithelium, was markedly overexpressed in K5-VILIP-1 animals (Figures 2D, E, F).

**Figure 3:**
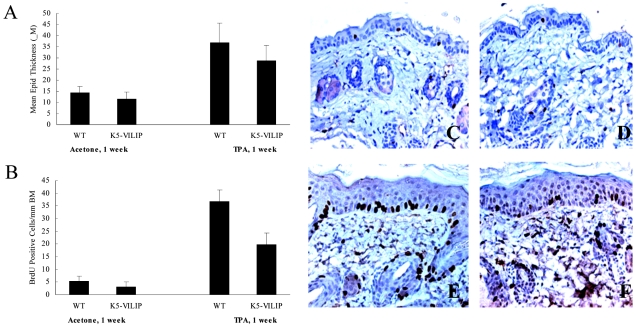
Effects of acute topical treatment with TPA. Mean epidermal thickness expressed in micrometers (A) shows a minor reduction of epidermal thickness in K5-VILIP-1 dorsal interfollicular epidermis with respect to WT epidermis (acetone treated controls) (p<0.02). After 7 days of TPA treatment (2 topical applications, at day 1 and day 4) there is an increase in epidermal thickness in both WT and transgenic mice. Note that this increase is significantly lower in transgenic epidermis (p<0.001). Panel B shows a proliferation index calculated as the number of BrdU positively stained basal keratinocytes per mm of basement membrane. Note significantly lower labeling index in control K5-VILIP-1 epidermis with respect to WT epidermis (p<0.002). Similarly, after TPA treatment the transgenic epidermis showed significantly reduced cell proliferation (p<0.0005). Note a general similarity in morphology between WT (C) and K5-VILIP-1 (D) dorsal epidermis stained with hematoxylin after acetone (control) treatment. Nevertheless, one week after TPA treatment, both WT epidermis (E) and K5-VILIP-1 epidermis (F) are thicker that their respective controls. Furthermore, WT epidermis treated with TPA (E) exhibits more BrdU-stained cells than the K5-VILIP-1 epidermis (F). BrdU immunohistochemistry counterstained with hematoxylin, X200.

The effect of VILIP-1 overexpression on epidermal thickness and basal keratinocytes proliferation was evaluated in hematoxylin and eosin (H&E) stained specimens and using BrdU immunohistochemistry respectively. A statistically significant difference was seen between the baseline epidermal thickness of WT and K5-VILIP-1 mouse epidermis in untreated mice. The measurements of the dorsal epidermal thickness in WT and transgenic mice indicated that epidermal thickness was slightly decreased by the expression of the transgene (p<0.02) (Figure 3A, left panel). Similarly, the labeling index of bromodeoxyuridine (BrdU) in basal keratinocytes (expressed as labeled basal cells per mm of basement membrane) showed that K5-VILIP-1 epidermis had a lower incorporation rate than the WT epidermis (42% decrease, p<0.005) (Figure 3B, left panel). Furthermore, decreased susceptibility to the hyperplasiogenic and proliferative effects of 12-0-Tetradecanoylphorbol-13-acetate (TPA) was observed after acute topical treatment. The epidermis from transgenic mice showed an approximately 22% decrease in thickness compared to their WT counterparts after one week of TPA treatment (Figure 3A, right panel). Proliferation was also evaluated by BrdU incorporation after TPA treatment. BrdU incorporation into K5-VILIP-1 epidermis decreased approximately 50%, with respect to the WT levels (Figure 3B, right panel; this can be visualized by comparing the micrographs in panels 3E and 3F).

### Overexpression of VILIP-1 decreases susceptibility to skin cancer

**Figure 4:**
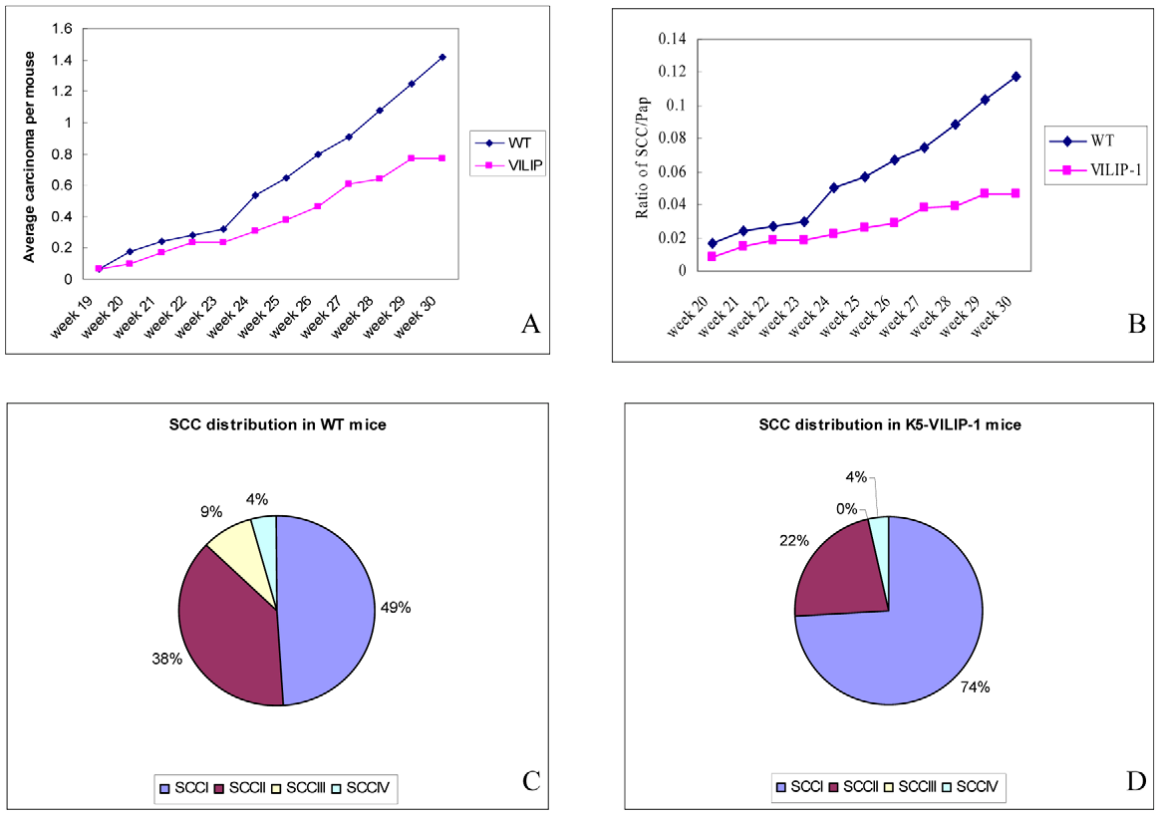
Responsiveness of WT and transgenic mice to two stage carcinogenesis protocol (DMBA/TPA). (A) Tumor multiplicity: SCC/mouse. Note decreased number of carcinomas in transgenic compared to WT animals (p<0.02). (B) SCC/papilloma ratio indicating a sharp decrease in K5-VILIP-1 with respect to WT mice (p<0.01). Histopathological grade is shown in Panels C and D: The relative distribution of SCCs according to their histopathological grade is depicted in the pie charts. Note the predominance of SCC I tumors in K5-VILIP-1 mice.

In order to determine the effects of VILIP-1 overexpression on SCC development, two different chemical carcinogenesis protocols were applied to the mouse skin. The two stage carcinogenesis protocol showed that K5-VILIP-1 mice had a 49% decrease in the SCC tumor multiplicity when compared to WT mice at week 30 (p<0.02) (Figure 4A). Interestingly, the ratio of SCC/papilloma was reduced more than two-fold in transgenic animals when compared to WT mice (Figure 4B) (p<0.01). This translates into a conversion rate of papillomas to carcinomas in this experiment of 23 for K5-VILIP-1 mice and 9 for WT mice. The transgenic mice showed a predominance of well differentiated SCC of grade I, 74% to 49% in WT mice (Figures 4C and 4D). Although not statistically significant, the tumor multiplicity of SCC I in K5-VILIP-1 mice was lower than in WT mice (0.68 SSCI/mouse versus 0.88 SCCI/mouse). When considering tumor multiplicity of higher grade tumors SCC II, III and IV there was a statistically significant difference, i.e. 0.08 SCCs/mouse in K5-VILIP-1 mice versus 0.31 in WT mice (p<0.003). Also of interest was the extremely low prevalence of high grade tumors in the K5-VILIP-1 mice, i.e., whereas in WT mice 13% of SCCs were of high grade (grades III and IV), in K5-VILIP-1 transgenic mice this proportion was only 4% (Figures 4C and 4D).

**Figure 5:**
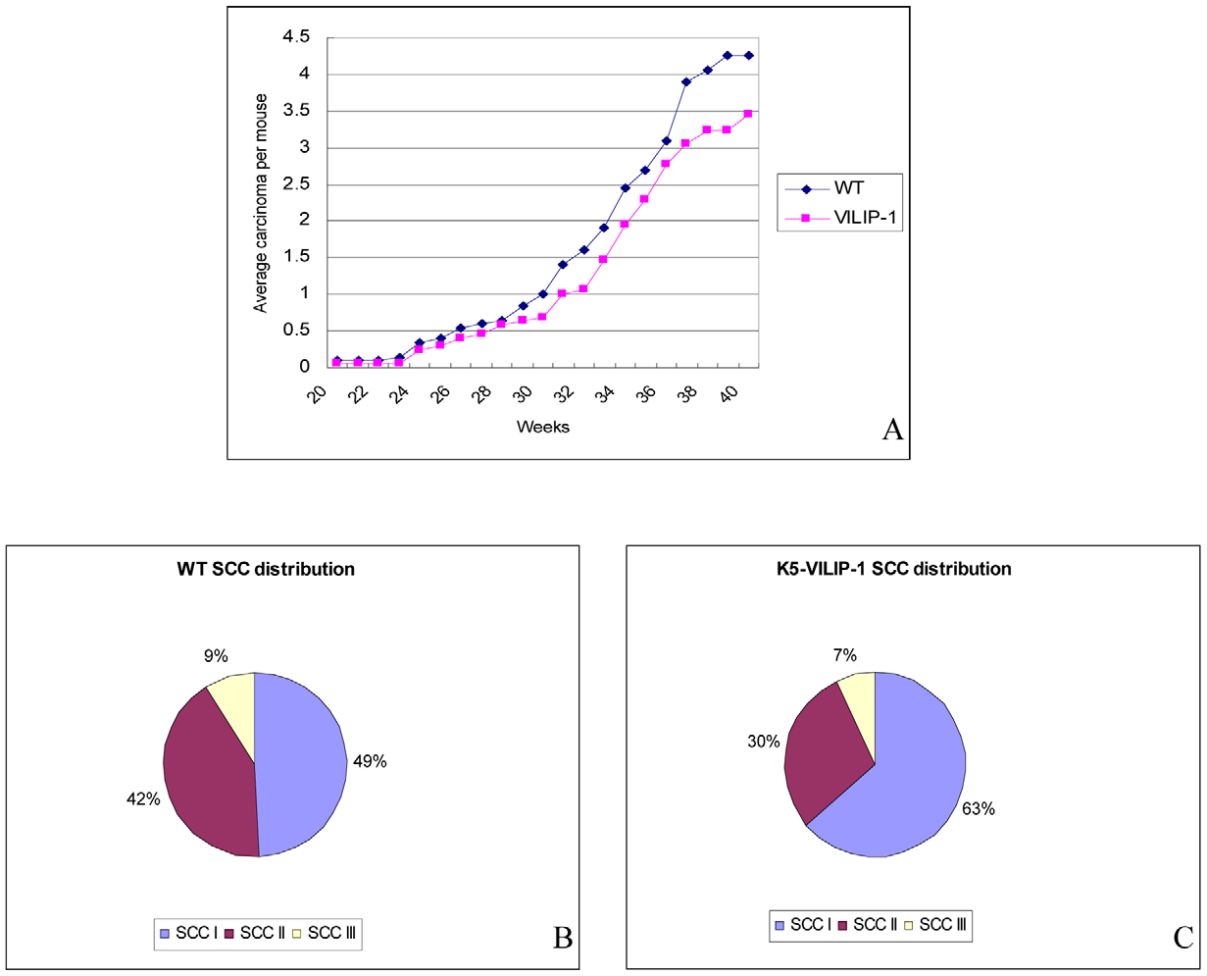
Responsiveness of WT and transgenic mice to complete carcinogenesis using B(a)P. (A) Tumor multiplicity: SCC/mouse. Note decreased number of carcinomas in transgenic compared to WT animals (p<0.05). Histopathological grade is shown in Panels B and C: The relative distribution of SCCs according to their histopathological grade is depicted in the pie charts. Note the predominance of SCC I tumors in K5-VILIP-1 mice.

The complete carcinogenesis protocol with B(a)P showed a similar tendency. Although with this protocol, the SCC multiplicity was higher than with the two stage carcinogenesis protocol, a reduction of approximately 20% was noted at the end of the experiment (between 35 to 40 weeks) in the K5-VILIP-1 with respect to WT mice (Figure 5A) (p<0.05). Histopathological analysis of the tumors at the final time point showed that there was an even larger difference when microscopic tumors, that were not detected grossly, were taken into account. This analysis showed that the reduction of SCCs was even higher (33%) in K5-VILIP-1 mice with respect to WT mice (p<0.05). The predominance of very well differentiated SCCs (Grade I) in transgenic mice, 63% up from 49% in WT mice, was noteworthy. The average number of high-grade tumors (II and III) per mouse was only 1 in K5-VILIP-1 transgenic animals as compared to 1.8 in wild-type mice (Figures 5B and 5C).

### Differences in tumor characteristics

**Figure 6:**
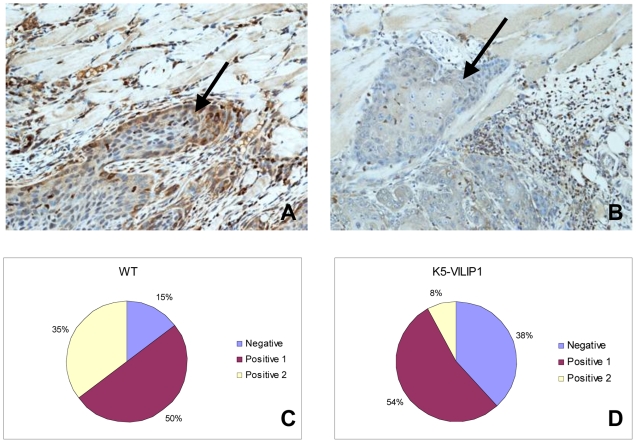
MMP-9 IHC analysis of the invasive front (arrow) of SCCs from a wild type mouse (A) and a K5-VILIP-1 (B) mouse treated with a two stage carcinogenesis protocol. The asterisk shows the location of the skin muscle layer. MMP9 immunohistochemistry with hematoxylin counterstain (X200). The pie charts show the percentage of SCCs exhibiting no immunostain (Negative), mild to moderate stain (Positive 1) and intense immunostain (Positive 2) in the WT (C) and transgenic (D) groups respectively.

The proliferation rate of papillomas produced by the two stage carcinogenesis protocol was determined by evaluating Ki67 expression of basal cells in WT and K5-VILIP mice at week 30. Interestingly the labeling index (LI) with this proliferation marker (percent of labeled cells stained in the basal layer) showed a significant decrease in LI of papillomas derived from transgenic mice with respect to similar benign tumors from WT mice (58% vs 76%, p = 7.04E-06). When the same comparison was done between SCCs from these two animal groups, a moderate but non-significant KI67 LI decrease in transgenic-derived malignant tumors was noted (52% vs 61%, p = 0.069). In addition, we noticed that the expression of MMP-9 was lower in transgenic-derived SCCs than in the equivalent tumors from K5-VILIP-1 mice (Figures 6A–D), i.e., almost 5 times as many SCCs from WT mice than SCCs from K5-VILIP-1 animals expressed high levels (Positive 2) of MMP-9 (35% WT vs 8% K5-VILIP-1, p <0.01).

## Discussion

VILIP-1 is expressed in the central nervous system, where it regulates cAMP levels, cell signaling and differentiation [5], [7], [8]. VILIP-1 is also widely expressed in sites outside the nervous system such as human heart, lung, liver and testis and moderately expressed in ovary, kidney, spleen and pancreas, suggesting that VILIP-1 might be required for the maintenance of tissue homeostasis in different organs [10]. Given the central role of VILIP-1 as a calcium sensor in mediating cAMP response, deregulation of VILIP-1 expression may cause abnormalities in various non-nervous tissues. In past reports, we have described that VILIP-1 expression is lost in chemically-induced mouse SCC [3]. We have also shown that VILIP-1 plays a critical role in regulating the invasive/metastatic phenotype by decreasing cell proliferation and matrix degradation/tumor cell invasiveness through a cAMP mediated pathway [3], [9]. Furthermore, we have found that VILIP-1 is lost in aggressive SCCs of the human esophagus and lung suggesting a tumor suppressor function [11], [12].

In this report we demonstrate that the transgenic expression of VILIP-1 targeted to the epidermis is able to decrease the baseline levels of cell proliferation and that this downregulation of epidermal proliferation is also very evident after short term treatment with the hyperplasiogenic tumor promoter TPA. The decrease in epidermal cell turnover is accompanied by an increased expression of products of keratinocyte differentiation, especially K1 and loricrin (Figure S1). The main effect on cell proliferation and differentiation is attributable to the well known increase in cAMP levels in keratinocytes overexpressing VILIP-1, described in detail in our previous reports [3], [9]. VILIP-1 expression also induced astrocytic differentiation in C6 cells [13] and is associated with increased squamous differentiation in human esophageal and lung tumors [11], [12]. In this context, it is noteworthy that Braunewell and Gundelfinger were able to demonstrate that differentiation is inducible using cAMP analogs [13]. Similar effects of cAMP have been described in squamous cell lines and tissues in which cAMP enhanced the expression of keratins such as K1 and K10 [14], [15]. Perhaps the most significant effect of VILIP-1 overexpression in epidermis is a decrease in MMP-9 activity. This was shown by us in VILIP-1 transfected cells [3] and was considered a consequence of increased cAMP activity [9]. In the present report we show that this decrease in primary keratinocytes derived from K5-VILIP-1 transgenic mice is accompanied by a significant increase in TIMP-1. This direct regulation of TIMP-1 levels by cAMP has been demonstrated in several different cells and tissue types [16], [17], [18], [19] and may be the principal mechanism of TIMP-1 induction by VILIP-1 overexpression in the transgenic mouse skin.

In order to evaluate whether VILIP-1 could modulate tumorigenesis and/or susceptibility to exogenous carcinogens we applied well known carcinogenesis protocols that are widely accepted as paradigms of epithelial carcinogenesis, i.e., the two stage carcinogenesis and the complete carcinogensis protocols of the mouse skin [20], [21], [22]. The decreased keratinocyte proliferation and increased squamous differentiation patterns observed in transgenic epidermis have a direct correlation with our observations during skin carcinogenesis of K5-VILIP-1 mice that led to a decreased sensitivity to skin carcinogenesis. In both carcinogenesis protocols we observed a decrease of SCC multiplicity with respect to WT mice that was close to 49% in the two stage carcinogenesis experiment during the final weeks of the experiment (28-30 weeks) and approximately 33% in the complete carcinogenesis experiment at 36 to 40 weeks. In the two stage carcinogenesis experiment it was noteworthy that the ratio of SCC to papillomas was markedly decreased in K5-VILIP-1, indicating that the conversion rate was diminished in transgenic mice. In addition to this general decrease in the prevalence of SCC in K5-VILIP-1 mice, we observed a remarkable change in the distribution of SCCs of different histopathological grades at the final time-point of the experiments. In both carcinogenesis protocols the transgenic mice had a predominance of low grade SCCs over high grade SCCs when compared with the respective WT treated mice. This was clearly represented by the increase in SCC I, i.e., very well differentiated SCCs, that constituted 74% of all SCCs in K5-VILIP-1 mice versus only 49% of SCCs in WT mice treated with the two stage carcinogenesis protocol. In the complete carcinogenesis experiment, the difference was slightly lower, 69% in K5-VILIP-1 mice versus 49% in WT mice. These changes in the histopathological grades correlate well with our data on differentiation patterns seen in the transgenic epidermis. VILIP-1 overexpression increases epidermal keratinocyte differentiation in TPA treated skin and this tendency may influence the predominance of well differentiated SCCs in transgenic mice. Decreased cell proliferation could also be an important factor because quite frequently well differentiated SCCs not only have more advanced differentiation patterns than high grade SCCs but are also characterized by a relatively slower cell proliferation [23], [24], [25]. This has also been seen in the tumors, especially in papillomas from transgenic K5-VILIP-1 animals, which showed a significant decrease in Ki67 labeling index with respect to the papillomas from WT mice. A similar tendency was seen in SCCs. Furthermore, MMP-9 expression in tumors from WT and transgenic animals showed the same pattern of MMP-9 activity seen in normal primary epidermal keratinocytes, i.e., tumors from transgenic origin showed less MMP-9 expression than that seen in tumors from WT mice treated with carcinogens.

Although, we evaluated local and distant metastases at the end of the experiments we did not find statistically significant differences (data not shown). This lack of differential metastatic abilities between SCCs from transgenic and WT mice is probably due to the fact that skin SCCs have relatively low and late metastatic potential [26], [27]. Furthermore, this inability to detect metastases is enhanced by the present bioethical guidelines that require culling animals that present with tumors larger than 10 mm diameter. The lack of significant metastases data notwithstanding, tumor grading evaluation indicated that the incidence of high grade SCCs, usually the most aggressive and invasive tumors, were decreased in K5-VILIP-1 transgenic mice. This data can be considered as evidence of decreased tumor progression in mice overexpressing this protein. This is supported by previous experiments demonstrating that overexpression of VILIP-1 in murine SCCs cell lines decreased invasion and migration [3].

In summary, transgenic VILIP-1 expression targeted to the epidermis results in decreased proliferation patterns that render the skin less susceptible to carcinogenesis. Furthermore, VILIP-1 overexpression attenuates the histotypes produced resulting in a slower conversion to overt malignancy in the two stage carcinogenesis protocol and a decreased prevalence of the most advanced high grade SCCs in both carcinogenesis protocols. These observations point to an inhibitory effect of VILIP-1 on tumor development, malignant conversion and on tumor progression.

## Materials and Methods

### Materials

12-0-Tetradecanoylphorbol-13-acetate (TPA), 7, 12-Dimethylbenz(a)anthracene (DMBA) and benzo(a)pyrene (B(a)P) were purchased from Sigma–Aldrich (St Louis, MO). FVB/N mice, 6-8 weeks of age, were purchased from Taconic (Germantown, NY) and were used as wild type controls.

### Generation and identification of K5-VILIP-1 mice

A similar approach to the one used previously in our laboratory to obtain transgenic mice was utilized [28]. The 0.85-kb full-length human *VILIP-1* cDNA (*VSNL1*) was excised from its parental pCIneo vector using DraI, which produces a blunt-ended fragment. The fragment was ligated into the *Sna*BI site between the rabbit β-globin intron and polyadenylation sequences from a vector described previously and inserted into the K5 expression vector [29]. Orientation and integrity of the insert were confirmed by restriction analysis and sequencing. The K5-VILIP-1 transgene was microinjected into the pronuclei of mouse embryos obtained from FVB female mice mated with FVB males. DNA was extracted from clipped tails as described [30]. Mice were genotyped by PCR analysis of tail DNA utilizing primers specific for β-globin intron and the sequences of primers were: sense, 5′TTCAGGGTGTTGTTTAGAAATGG; antisense, CAATAAGAATATTTCCACTCCA. All research involving animals was conducted according to the relevant national and international guidelines. All animals were kept in isolator cages in a pathogen-specific free environment. All mice used in these experiments were maintained on a 12:12 h light: dark cycle. Mice were given food and water ad libitum throughout the experimental period. Tumor-bearing mice were euthanized when the tumor was larger than 12 mm diameter, ulcerated or the animal had clear signs of discomfort or disease (rapid weigh loss, weakness or listlessness). The mice were cared for in accordance with the Guide for the Care and Use of Laboratory Animals and the experimental protocols were annually approved by the Institutional Animal Care and Use Committee Fox Chase Cancer Center (protocol No. 99-14). All animal experiments at our Institution must obtain such an approval (our facility is ALALC approved).

### Southern Blot analysis of copy number

10 µg of liver DNA was digested with EcoRI, and then subjected to electrophoresis on 0.8% agarose and transferred to Hybond N^+^ (Amersham, Buckinghamshire, UK). The blot was hybridized with a probe from transgene labeled by PrimerIt II Random Primer Labeling kit (Stratagene, La Jolla, CA). The transgene band to be detected is a 700-bp DNA fragment generated by EcoRI digestion of chromosomal DNA. As a standard for determining the copy number we digested the pBSK5-VILIP-1 plasmid used for the microinjections with EcoRI, separated by 1% agarose gels and the corresponding 700 bp band was excised and purified. The amount of controls was calculated as describe in http://www.med.umich.edu/tamc/spike.html. Estimation of the copy number of the transgene was done by densitometry, comparing the intensity of the signals generated by hybridization of the ^32^P-dCTP-labeled probe with DNA extracted from transgenic animals with those generated by controls containing 0, 1, 3, 9 and 18 copies of the 700-bp band per genome.

### Culture of newborn keratinocytes and Western blot analysis of VILIP-1 expression

Primary epidermal keratinocytes from newborn mice were used to determine expression levels of VILIP-1 because of their suitability for *in vitro* growth and further molecular analyses. Primary epidermal keratinocytes were established *in vitro* as described [31], [32]. Briefly, 1–3 days-old mice were sacrificed; the skin was washed in a 1:10 solution of Betadine, rinsed twice in sterile water and twice in 70% alcohol. The skin was removed and floated overnight on 2 ml of trypsin (0.25% without EDTA). The epidermis was separated from the dermis, minced and resuspended in HiCa medium containing calcium-free MEM Eagle (#06-174G, Lonza, Walkersville, MD), 8% chelexed serum (provided by Cell Culture Facility, Fox Chase Cancer Center) and 1.3 mM calcium. The resulting cell suspension was triturated by pipetting up and down and applied to 100-µm cell strainer (BD Biosciences, Bedford, MA). After centrifugation, the pellet was resuspended in 0.2 mM calcium medium (calcium-free MEM Eagle, 8% chelexed serum and 0.2 mM calcium) and counted. The cells were initially plated in 0.2 mM calcium medium for 1 day, and then plated in KGM growth medium composed of 1 part of KBM Basal Medium (#CC-3101), 2 parts of KBM Basal Medium without calcium (#CC-3104) and bovine pituitary gland extract (from #CC-4131). The keratinocytes grown in culture dishes were lysed and subjected to VILIP-1 Western blot analysis according to our previously reported protocol [12].

### Mouse cAMP assay

Mouse primary keratinocytes were seeded onto 96-well culture plate and allowed to grow till at least 70% confluency was reached. The cells were lysed and the intracellular cAMP concentration was measured using the cAMP Biotrak Enzymeimmunoassay (EIA) system (#RPN2251, Amersham Biosciences Corp, Piscataway, NJ) following the manufacturer's instructions. The relative amount of the intracellular cAMP (fmol) from VILIP-1 transgenic keratinocytes was normalized with respect to the values obtained from wild type keratinocyte cultures.

### Mouse TIMP-1 assay

About 10 million mouse keratinocytes per 10-cm culture dish were seeded. After 2 days in culture, the cells were washed twice with PBS (calcium and magnesium free) and maintained in 10 ml conditioned medium containing 1 part of KBM Basal Medium (#CC-3101) and 2 parts of KBM Basal Medium without calcium (#CC-3104) (Lonza, Walkersville, MD) for 1 day. The volume of the conditioned medium was reduced to 150 µl by using Amicon Ultra centrifugal filters (10k, #UFC901024) and Microcon YM-10 (#42407) (Millipore, Billerica, MA). The concentration of TIMP-1 in conditioned medium was measured using RayBio mouse TIMP-1 ELISA kit (#ELM-TIMP1-001, RayBiotech Inc., Norcross, GA). Relative concentration of TIMP-1 (pg/ml) in conditioned medium from VILIP-1 transgenic keratinocytes was normalized with respect to the values obtained in conditioned medium from wild type keratinocyte cultures.

### Mouse MMP-9 assay

MMP-9 activity was determined by gelatinase zymography. 5 µl of conditioned medium was mixed with 5 µl of Novex Tris-Glycine SDS Sample Buffer (2x, #LC2676, Invitrogen, Carlsbad, CA) and loaded on Novex 10% Zymogram (Gelatin) Gel (#EC6175Box). The samples were fractionated in 1x Novex Tris-Glycine SDS Running Buffer (#LC2675) for 90 min at 120V. After electrophoresis, the gel was incubated in Zymogram Renaturing Buffer (#LC2670) for 30 minutes and thereafter in Zymogram Developing Buffer (#LC2671) overnight. The gel was finally stained with Colloidal Blue Staining Kit (#LC6025) to visualize the areas of protease activity.

### Tumor induction experiments

#### Two stage carcinogenesis protocol

A single 100-nmol initiating dose of DMBA in 0.2 ml acetone was applied topically to shaved dorsal skin of 6–8 week-old female mice. One week after DMBA treatment, TPA (4 µg) in 0.2 ml of acetone or acetone alone was applied twice weekly to the skin for the duration of the experiment (30 weeks). Tumor incidence and multiplicity were observed weekly starting at 8 weeks of TPA promotion. The number of mice per group was as follows: 28 wild-type mice, 30 K5-VILIP-1. Carcinomas were recorded by gross observation as infiltrating and/or ulcerating lesions and confirmed by histological analysis (see below). Autopsies of carcinoma-bearing mice were performed and metastasis in axial lymph nodes, lung, liver and spleen were recorded. All tumors were analyzed histologically. Squamous cell carcinomas (SCC) were classified according to histopathological grade [33] . Most SCCs were endophytic growths that invaded the dermis and subcutaneous tissue. The differentiation patterns defining the histopathological grade were: a) Grade I SCC: Very well-differentiated with most of the tumor containing keratinizing cells and horny pearls, b) Grade II SCC: Moderately differentiated tumors in which up to 50% of the tumor mass is formed by keratinizing cells c) Grade III SCC or poorly differentiated tumors: Containing less than 25% tumor mass showing evidence of keratinization, and d) Grade IV SCCs: Very poorly differentiated tumors or spindle cell carcinomas containing very little or no histological evidence of keratinization. Papilloma and carcinoma were photographed at a magnification of 2.5× (NA 0.08) and 10× (NA 0.45) respectively.

#### Complete carcinogenesis protocol

18 wild-type and 16 K5-VILIP-1 female mice were treated with 0.15 µmole (B(a)P) twice weekly for 35 weeks in disposable cages, processed and evaluated as described above for two stage carcinogenesis.

### Analysis of epidermal thickness and cell proliferation following treatment with TPA

Groups of wild-type or K5-VILIP-1 transgenic mice (*n* = 5) were treated with two applications of TPA (5 nmol) or the acetone vehicle and sacrificed 48 hours after the last treatment. Untreated mice were also included as control. Paraffin sections were stained with hematoxilin and eosin (H&E) and the skin thickness was measured with a micrometer. Ten measurements per mouse were done in the interfollicular epidermis every 150 micrometers. For analysis of cell proliferation, mice treated as described above, were injected intraperitoneally with BrdU (100 µg/g body weight, Sigma-Aldrich) in PBS, 2 hours prior to sacrifice. Treated skin was then fixed in formalin, embedded in paraffin, sectioned at 4 µm, stained with H&E and anti-BrdU antibody (Roche) (dilution 1/100), and then treated with biotinylated anti-mouse IgG and HRP-conjugated ABC reagent (Vector Laboratories Inc., Burlingame, CA). Slides were mounted and observed with a Nikon Optiphot with a Plan/Apo objective 20X, NA, 0.75, Nikon eyepiece X10, final magnification ×200. Epidermal cell proliferation (presented as BrdU labeled cells/mm of basement membrane) was determined as follows: Basement membrane (BM) length was determined in skin sections and the corresponding number of BrdU labeled cells of the respective interfollicular epidermal sector was counted. BM length determinations were done with the aid of the Image Pro-Plus imaging software (Media Cybernetics, Silver Spring, MD). BM length was determined in acetone and TPA treated epidermis (3-5 mice per group, minimal BM length/mouse was 2 mm per section).

### Immunohistochemistry

Immunohistochemical procedures were performed on formalin-fixed and paraffin-embedded dorsal skin and skin tumors. All paraffin sections were subjected to previously published immunostaining protocols [33]. The same anti VILIP-1 antibody used in Western blot analysis was employed as primary antibody at 1/500 and 1/1000 dilutions. An avidin-biotin-peroxidase kit (Vectastain Elite, Burlingame, CA) was then employed followed by the chromagen 3′3′-diaminobenzidine to develop the immunostain. Negative controls, not incubated with VILIP-1 antibody, were incubated with pre-immune serum at the same concentrations as the primary antibody. Ki67 (Vector Labs., Burlingame, CA, rabbit polyclonal VP-K451, 1/6000 dilution with antigen retrieval) and MMP-9 (R&D Systems, Minneapolis, MN; goat anti mouse MMP-9, AF909) were detected in tumors by immunohistochemistry using similar procedures. Ki67 labeling index was determined in the basal layer of papillomas (n = 22) or basaloid SCC cells (defined as carcinoma cells in contact with stroma) (n = 20) from the two stage carcinogenesis experiment by counting at least 400 cells per tumor. MMP-9 was evaluated in twenty SCCs from the two stage carcinogenesis experiment using a semiquantitative scale (Negative: no stain; positive 1: moderate stain, mostly focal in nature, less than 50% of the tumor cells; positive 2: intense stain, more than 50% of the tumor cells). All sections were counterstained with hematoxylin.

### Statistical Analysis

Statistical significance was determined by calculating P values. P values corresponding to the curves of carcinoma development were determined two-tail T tests.

## Supporting Information

Figure S1 Differentiation patterns in TPA treated epidermis. After 2 topical applications of TPA the epidermis shows increased thickening in both WT and K5-VILIP-1 mice. K1 and loricrin (LOR) are clearly overexpressed in K5-VILIP-1 epidermis with respect to WT epidermis (panels A-B and G-H). K6 and K14 are marginally increased or unchanged when compared with transgenic epidermis (panels C-D and E-F). Marker immunohistochemistry and hematoxylin counterstain, X200.
(3.45 MB TIF)
