# Supplementary figures and images for: VILIP-1 Expression In Vivo Results in Decreased Mouse Skin Keratinocyte Proliferation and Tumor Development
Source: PLoS One. 2010 Apr 15;5(4):e10196. doi: 10.1371/journal.pone.0010196 (PMC2855367; doi:10.1371/journal.pone.0010196)

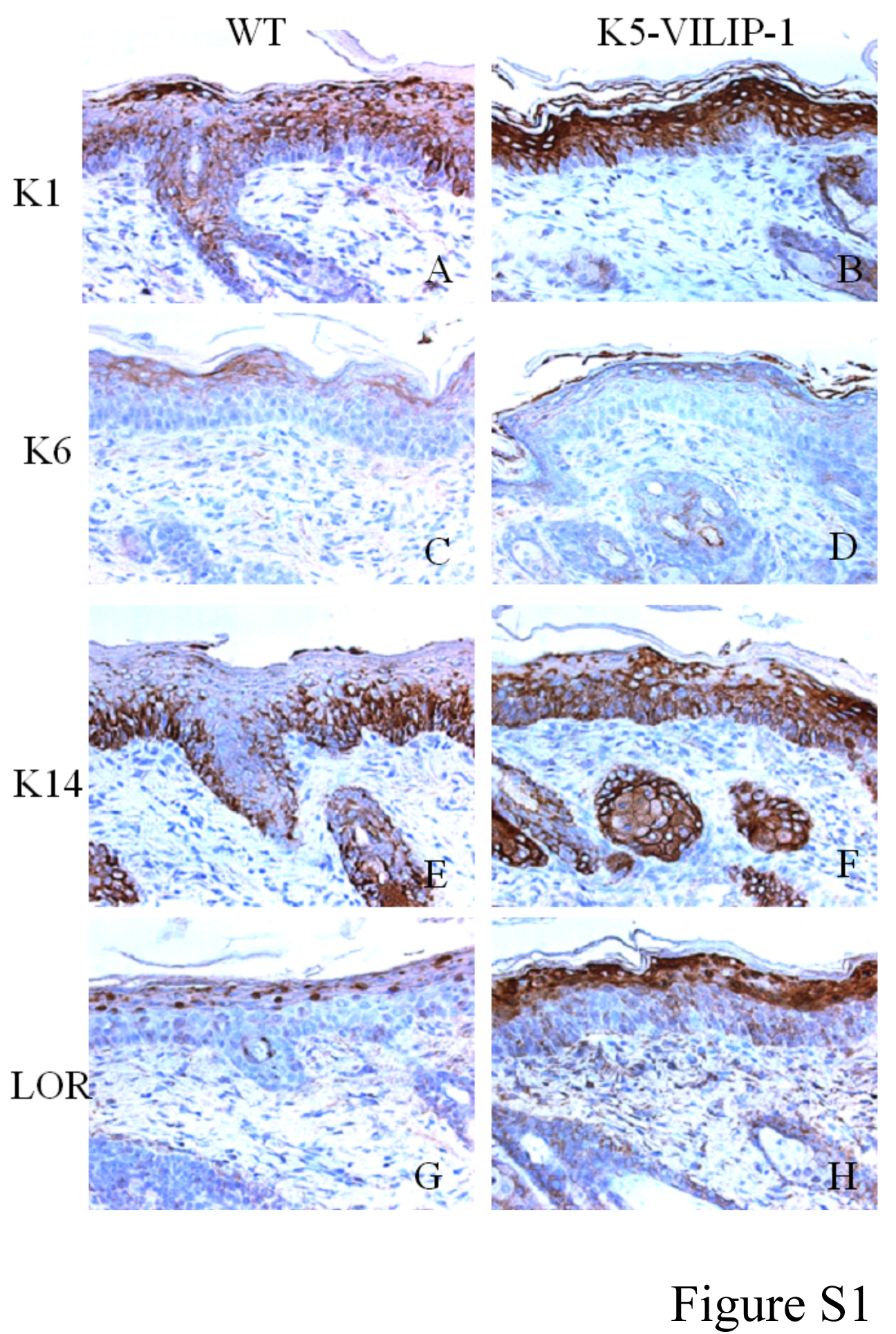

Supplement: Figure S1 — Differentiation patterns in TPA treated epidermis. After 2 topical applications of TPA the epidermis shows increased thickening in both WT and K5-VILIP-1 mice. K1 and loricrin (LOR) are clearly overexpressed in K5-VILIP-1 epidermis with respect to WT epidermis (panels A-B and G-H). K6 and K14 are marginally increased or unchanged when compared with transgenic epidermis (panels C-D and E-F). Marker immunohistochemistry and hematoxylin counterstain, X200. (3.45 MB TIF) [file pone.0010196.s001.tif]
